# Integrating a Fragmented Pharmacy Network into a Unified Supply Chain System: A Case Study from Qatar’s Primary Healthcare

**DOI:** 10.2147/JHL.S576024

**Published:** 2026-06-29

**Authors:** Manal Al-Zaidan, Chiragkumar Gohel, Regina Padmanabhan, John Gibb, Rafeeq Badiger, Mohamed Ghaith Al-Kuwari, Laoucine Kerbache

**Affiliations:** 1 Department of Pharmacy and Therapeutics Supply, Primary Health Care Corporation, Doha, Qatar; 2 College of Science and Engineering, Hamad Bin Khalifa University, Doha, Qatar; 3 Pharmacy and Drug Control Department, Ministry of Public Health, Doha, Qatar; 4 Department of Strategy Planning and Health Intelligence, Primary Health Care Corporation, Doha, Qatar; 5 Enterprise Data Warehouse, Health Information & Communication Technology, Primary Health Care Corporation, Doha, Qatar; 6 College of Medicine, Qatar University, Doha, Qatar

**Keywords:** pharmaceutical supply chain, enterprise data warehouse, business intelligence, enterprise resource planning

## Abstract

**Introduction:**

Healthcare organizations increasingly rely on digital integration to strengthen pharmaceutical supply chain governance. At the time of this study, Qatar’s Primary Health Care Corporation (PHCC) operated a decentralized pharmacy network without a dedicated internal Enterprise Resource Planning (ERP) system, resulting in fragmented procurement data and limited enterprise-level visibility.

**Objective:**

This study aimed to design and implement an enterprise-level data integration and analytics solution to enhance procurement transparency, inventory visibility, and data-informed decision-making across PHCC’s pharmacy network.

**Methods:**

Guided by a Design Science Research (DSR) methodology, a centralized Enterprise Data Warehouse (EDW) was developed by integrating procurement data extracted from the supplier’s ERP system. A Tableau-based analytics dashboard featuring key performance indicators (KPIs) was designed and deployed across 32 pharmacies to support operational monitoring and governance.

**Results:**

The implemented solution enabled real-time visibility of pharmaceutical procurement and inventory performance. Descriptive operational indicators showed a reduction in back-order rates from 6.7% to 4.5% in 2024. Inventory indicators demonstrated an increase in average medication shelf life from 551 to 568 days (3%) and an improved expiry profile, with 96% valid inventory and only 3% very short-expiry items compared with historical levels.

**Conclusion:**

This case study demonstrates how integrating external supplier ERP data into an enterprise data warehouse can enhance visibility, transparency, and decision support in public healthcare systems lacking an internal ERP. The approach provides a scalable and practical model for strengthening pharmaceutical supply chain governance without large-scale ERP implementation.

## Introduction

Healthcare organizations face increasing pressure to optimize their supply chain operations while maintaining high standards of patient care. Pharmaceutical procurement represents a significant portion of operational costs and directly impacts medication availability and patient outcomes.^1^ Despite the critical nature of medication management, many healthcare organizations struggle with limited visibility into their procurement processes due to data silos and inadequate analytics capabilities.

**Table 1:** Key Implementation Challenges and Solutions

	Challenge	Solution
**Data Access Constraints**	The supplier’s ERP system had limited external access capabilities and strict security protocols.	We developed ETL Pipeline using Microsoft SSAS to incrementally load the data in PHCC EDW
**Data Quality Issues**	Raw ERP data contained inconsistencies in product coding, units of measure, and transaction classifications.	We implemented robust data cleansing process within the Pipeline validating the data by applying business rules and exception handling to ensure data integrity
**Performance Optimization**	Initial dashboard performance was suboptimal due to the volume of data and complexity of calculations.	We implemented data aggregation in SSAS Model to optimize and improve performance while Tableau dashboard is refreshed to reflect current data daily.

Healthcare spending in Qatar is among the highest in the region, despite the country’s relatively small population.^2^ Qatar’s pharmaceutical market is projected to grow by 70% from 2020, reaching QR 7.5 billion by 2025, driven by regulatory reforms, digital health initiatives, and a national push for self-sufficiency and supply chain resilience.^3^

Key pharmaceutical providers in Qatar include Hamad Medical Corporation (HMC) and the Primary Health Care Corporation (PHCC), with HMC overseeing the public provision of pharmaceutical items. The PHCC, in turn, relies on HMC for the procurement, storage, and distribution of medications to its health centers (HCs).^4^

The Qatar’s National Health Strategy 2024–2030 places significant emphasis on enhancing Health System Efficiency and Resilience, aiming to address key challenges within Qatar’s healthcare sector. This priority focuses on improving governance, system integration, and digitization to optimize the use of resources, increase system sustainability, and foster innovation. With limited data availability and low adoption of digital solutions across providers, a key challenge remains the need for a robust, data-driven decision-making framework.^5^

### Literature Review

#### Healthcare Supply Chain Management

Settani et al^6^ conducted a systematic review of pharmaceutical supply chain models from an operations research perspective, highlighting the need for systems thinking approaches to address the complexity of healthcare supply networks Their research identified several key factors that influence pharmaceutical supply chain performance, including inventory policies, distribution network design, and demand forecasting accuracy.

The pharmaceutical supply chain presents unique challenges compared to other industries due to regulatory requirements, product perishability, and direct impact on patient outcomes. Rajabi et al^7^ proposed optimization strategies for pharmaceutical inventory management that consider both hospital and pharmaceutical company perspectives Similarly, Kelle et al^8^ examined specific inventory solutions for hospital pharmaceutical supply chains, emphasizing the importance of integrated approaches.

Fernandez et al^9^ developed a predictive decision support system for hospital inventory management that combines statistical modelling with healthcare-specific operational constraints Their model demonstrated significant reductions in both stockouts and carrying costs by integrating supplier data with internal systems to improve forecasting accuracy, particularly valuable for decentralized healthcare environments.

Ahmadi et al^10^ conducted a comprehensive literature review on inventory management of surgical supplies and sterile instruments in hospitals, identifying key challenges such as demand variability, product expiration, and storage constraints. Their research highlighted the importance of evidence-based approaches to inventory management in healthcare settings to reduce costs while ensuring product availability.

#### Enterprise Data Warehousing and Business Intelligence in Healthcare

The implementation of enterprise data warehousing (EDW) and business intelligence (BI) systems in healthcare has been recognized as essential for improving operational efficiency and decision-making. Batz et al^11^ proposed a framework for BI implementation in healthcare that addresses the unique challenges of the healthcare context, including data complexity, privacy concerns, and organizational barriers.

Healthcare organizations generate vast amounts of data across different systems, creating opportunities for analytics-driven insights. Shahzad et al^12^ explored the capabilities and potential benefits of big data analytics for healthcare organizations, identifying opportunities for improved operational efficiency, clinical decision support, and strategic planning. However, they also noted significant challenges related to data integration, quality, and security. The potential of big data analytics in healthcare extends beyond operational improvements to clinical outcomes. They emphasized the importance of addressing technical, organizational, and governance challenges to realize the full potential of healthcare analytics.

#### Enterprise Resource Planning (ERP) Implementation in Healthcare

Studies discuss key factors affecting successful implementation of hospital information systems, including user involvement, top management support, and adequate training programs.^13^ Their findings emphasized the importance of considering both technical and organizational factors during implementation. Butarbutar et al^14^ conducted a systematic literature review of ERP post implementation and identified active user participation, top management support, and training as three of the most critical success factors, emphasizing that organizational and environmental drivers often outweigh purely technical ones in determining ERP outcomes.

Al-Assaf et al^13^ conducted a stakeholder centric case study in the UAE healthcare sector, showing how physicians, administrators, and IT staff each exert distinct forms of influence, and how mapping those power relations and tailoring communication and governance structures accordingly can avert resistance and drive adoption. Their work demonstrates that proactive stakeholder engagement and aligning diverse interests through formal change management processes are key to ERP success in hospital settings.

Stefanou et al^15^ analyzed ERP system implementation in a hospital through a stakeholder analysis approach, highlighting the importance of understanding the diverse interests and power dynamics among different stakeholder groups. Their research demonstrated how stakeholder analysis can help identify potential sources of resistance and develop appropriate implementation strategies. They presented the case study of healthcare environment, detailing the challenges and success factors in connecting ERP systems with clinical information systems. They emphasized the importance of careful planning, user involvement, and phased implementation approaches in healthcare settings.

A recent empirical study by Bialas et al^16^ investigated the digitalization of healthcare supply chains through ERP systems adoption in hospitals. The study demonstrated a statistically significant association between the use of ERP systems and hospital supply chain costs. They found that technological and organizational readiness, hospital size, governmental policies, and perceived benefits significantly influence the extent of ERP adoption. Their findings align with our approach, highlighting that supply chain costs account for 40–50% of a healthcare provider’s total costs, suggesting that ERP-driven supply chain performance improvement could substantially increase operational efficiency and reduce costs.

#### Performance Measurement and Dashboards

A balanced scorecard approach for supply chain performance measurement that considers financial, customer, internal business process, and innovation perspectives helps organizations align operational metrics with strategic objectives. Gazisaeidi et al^17^ and Coiera et al^18^ identified key practical issues in the development of performance dashboards in the healthcare sector, including data quality, user interface design, and alignment with organizational goals. Their research provided practical guidelines for implementing effective dashboards in healthcare environments. Coiera et al^18^ and Helminski et al^19^ examined the use of quality dashboards by hospital boards, finding that well-designed dashboards can improve governance and strategic decision-making. Their research highlighted the importance of selecting meaningful metrics and presenting them in ways that facilitate understanding and action.

Lemak et al^20^ interviewed 51 CEOs and CIOs across 33 U.S. health systems and found that leaders who actively champion interactive dashboards and Electronic Health Record (EHR) tools achieve stronger alignment of their information systems with strategic priorities and report more successful strategy execution. They also noted persistent challenges around integrating disparate data sources, ensuring data quality, and maintaining robust security in complex digital environments.

The potential of big data analytics in healthcare extends beyond operational improvements to clinical outcomes. Polimenu et al^21^ explored the promise and potential of big data analytics in healthcare, highlighting applications in genomics, clinical decision support, and population health management. They emphasized the importance of addressing technical, organizational, and governance challenges to realize the full potential of healthcare analytics.

Ghazisaeidi et al^17^ investigated how CEOs use management information systems for strategy implementation in hospitals, finding that interactive use of information systems is associated with more successful strategy implementation.

Recent research has highlighted the importance of performance measurement in pharmaceutical supply chains and implementing structured balanced scorecard approaches for supply chain management.^22^,^23^ Similarly, Hjelle et al^24^ demonstrated the value of dashboards in performance management, showing significant improvements in decision-making processes. Settanni et al^6^ provide a comprehensive systems view of pharmaceutical supply chain models Ahmadi et al^10^ identify key inventory management practices for healthcare supplies.

### Background and Motivation

**Figure 1:**
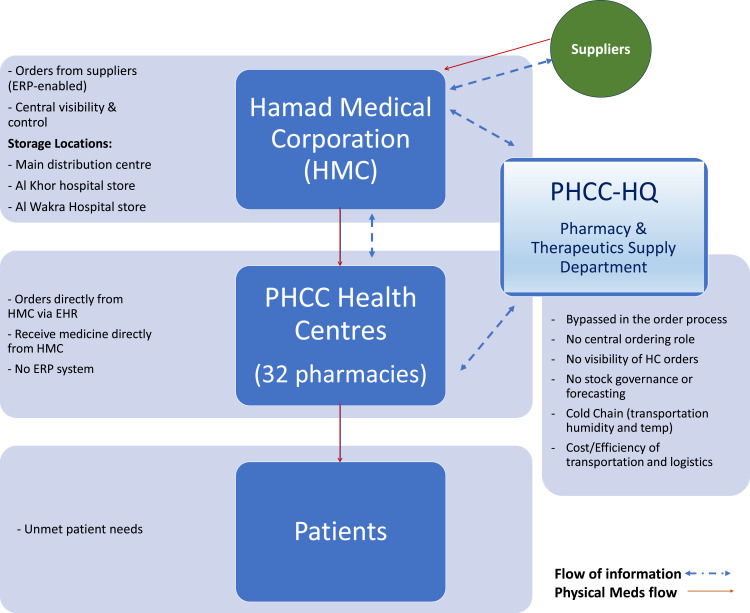
The Current Pharmaceutical Supply Chain Network Design.

Our healthcare organization comprises a network of 32 pharmacies distributed across primary healthcare centers throughout the State of Qatar. While each pharmacy operates within its local EHR environment to serve immediate clinical needs and procurement activities, there was no centralized monitoring or governance system at the corporate headquarters level. As a result, corporate leadership lacked real-time visibility into inventory status, procurement performance, and expiry risk across the network. This decentralized structure, combined with the lack of an enterprise-wide ERP system to manage pharmaceutical supply chain, created significant visibility challenges for leadership and prevented standardized inventory management practices across locations. (Figure 1)

The pharmaceutical supply chain at the Primary Health Care Corporation (PHCC) in Qatar currently operates through a semi-digital, partially integrated model that supports medication distribution across 32 health centers. The system leverages PHCC’s EHR platform, and the supplier’s ERP system, which are integrated solely for the purpose of transmitting medication requests. However, PHCC does not have its own ERP system, and all downstream supply chain processes such as approval, allocation, inventory tracking, and reconciliation are managed manually, resulting in a high volume of manual transactions and duplicated effort across sites.

The workflow begins at the healthcare center (HC) pharmacy, where medication requests are initiated via the EHR. Through the existing one-way integration, these requests are transmitted directly into the supplier’s ERP system for processing. In parallel, the same requests are exported as PDF files and manually emailed to the Pharmacy and Therapeutic Supply (PTS) team, which is based at the supplier’s distribution center (DC). Despite being physically located at the supplier site, the PTS team does not have system access to the supplier’s ERP system, nor a position in the PHCC’s EHR system. As a result, review and approval of requests, as well as any decisions regarding inter-pharmacy stock reallocation, are performed manually using Email attachments and internal communication, creating delays, limited traceability, and increased administrative workload for pharmacists and supply staff.

A key responsibility of the PTS team is to assess whether requested medications can be reallocated between HCs to reduce overstocking and minimize waste, particularly for items nearing expiry. However, the absence of consolidated inventory visibility restricts proactive reallocation decisions, and assessments are dependent on manually shared information. Once the manual review is completed, and no reallocation is needed, the supplier proceeds to fulfill the request through its ERP system. Items are picked, verified, packed with a delivery note, and dispatched to the requesting HC.

Upon delivery, the HC pharmacy lead or delegate verifies the shipment and acknowledges receipt in the EHR within 24 hours. The items are then stocked and made available for dispensing. Medication requests from clinical units (eg., nursing staff) are submitted through the EHR system and fulfilled by the HC pharmacy, completing the cycle. While this process ensures continuity of medication supply, it exposes patients to potential delays during procurement bottlenecks and limits pharmacists’ ability to focus on clinical services.

While this model offers basic centralized oversight and ensures medication availability, it is hampered by several critical limitations. The lack of a PHCC-owned ERP system prevents full integration and automation of inventory, procurement, and stock monitoring functions. All approvals, reallocations, and reconciliations are conducted manually, introducing variability, inefficiencies, and risks of miscommunication. Email remains the primary coordination tool, which is inherently prone to delays, data fragmentation, and tracking difficulties. Additionally, the system lacks real-time inventory visibility, predictive analytics, and automated alerts, making it difficult to proactively manage expiries, forecast demand, or optimize inventory levels across the 32 sites.

**Figure 2:**
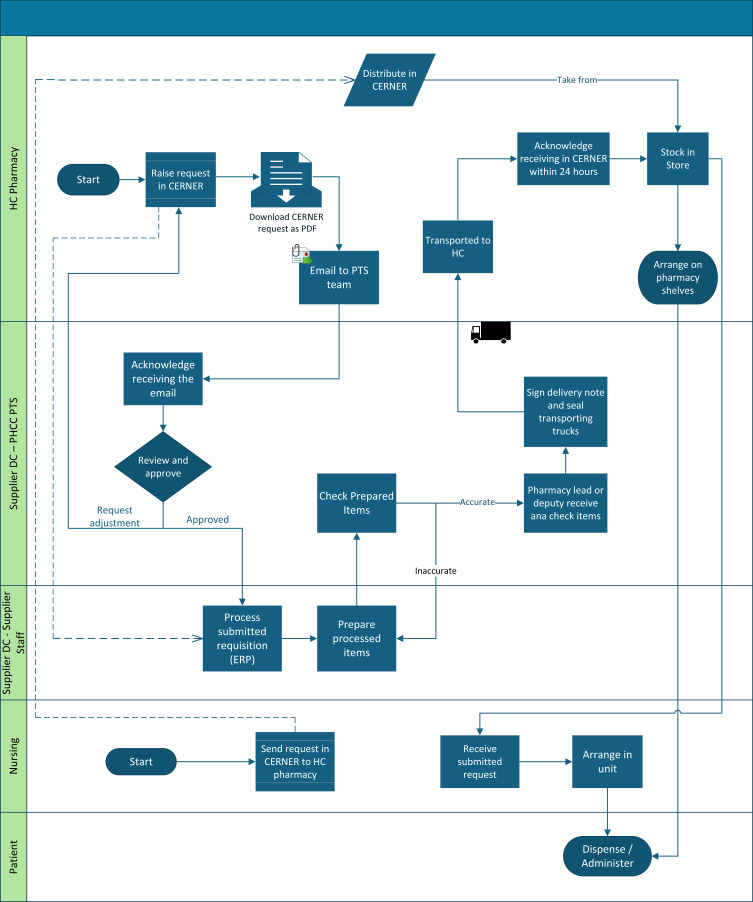
The Current Pharmaceutical Supply Chain Workflow.

These structural and technological gaps highlight the pressing need for a comprehensive and digitally integrated pharmaceutical supply chain model tailored to PHCC. Such a system would enhance transparency, reduce waste, support data-driven decision-making, and ensure a more resilient and efficient medication supply process (Figure 2).

Without a dedicated ERP system specifically designed for medication management, the organization faced several operational and safety challenges. These included the inability to accurately track medication expiration dates, which increased the risk of waste and potential medication errors. There was limited visibility into lot and batch numbers, complicating recall management and regulatory compliance. Discrepancies between physical inventory and system records necessitated frequent manual reconciliation, while procurement processes remained inefficient due to the absence of integrated supply chain data. Decision-makers lacked timely, consolidated information to support system-wide planning and governance. Additionally, the organization struggled to forecast demand accurately and optimize inventory levels. The lack of standardized inventory practices across the 32 pharmacy locations further hindered efficiency. Corporate leadership also lacked the tools to effectively monitor and govern pharmacy operations, and the fragmented nature of data made it difficult to implement system-wide quality improvement initiatives.

The supplier organization, however, utilized a comprehensive ERP system that contained valuable procurement data including complete lot/batch tracking, expiration date management, and detailed transaction history. This presented an opportunity to address internal ERP limitations by leveraging external ERP data through enterprise level integration and analytics.

To address the challenges, we initiated a project collaborating with PHCC EDW team to integrate data from the supplier’s ERP system into PHCC Enterprise Data Warehouse and built a Data mart (Tabular Model) using Microsoft SQL Server Analysis Services (SSAS). Key performance indicators (KPIs) were designed to enable enterprise-wide monitoring, analytics, and governance of pharmaceutical procurement and inventory. This approach would complement our existing EHR system by providing a missing pharmaceutical supply chain capabilities without requiring large-scale ERP implementation within PHCC. It also enabled centralized visibility across all 32 pharmacies, supporting standardized practices and system-wide quality initiatives.

The integration effort aligned with broader organizational goals to enhance medication safety, reduce waste through better expiration date management, improve regulatory compliance through enhanced lot/batch tracking, and increase operational efficiency through data-driven inventory management. Similar challenges are reported across public healthcare systems internationally, particularly where organizations rely on external suppliers or legacy systems rather than integrated ERP platforms, reinforcing the broader relevance of this case. By accessing supplier ERP data and transforming it into actionable insights, the project aimed to bridge the gap between clinical operations and supply chain governance.

This paper aims to describe the integration of supplier ERP data into a centralized enterprise data warehouse and Tableau dashboard for real-time visualization of pharmaceutical procurement, addressing a documented gap in empirical evidence on how public healthcare systems can integrate external ERP data to achieve enterprise-level visibility without an internal ERP system, and to support strategic and operational decision-making.

### Ethics Approval

The data analyzed in this study were obtained from the Primary Health Care Corporation (PHCC) Tableau dashboard, which provides aggregated operational data accessible within the organization. The dataset did not include any patient-identifiable information. Accordingly, this study was considered exempt from Institutional Review Board (IRB) approval.

## Methods

**Figure 3:**
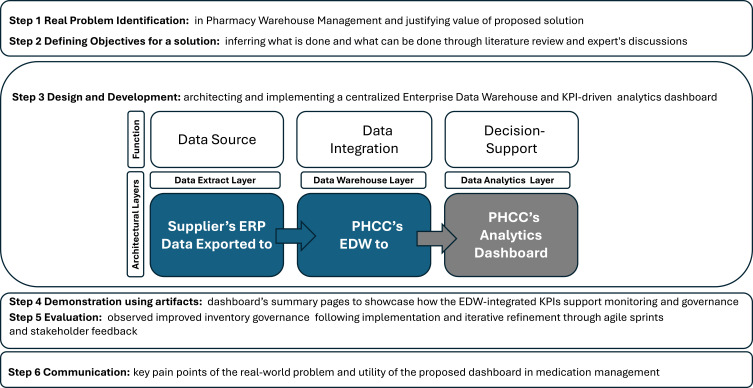
DSR Process Model.

This study adopted a Design Science Research (DSR) methodology as shown in Figure 3 to address limited pharmaceutical procurement visibility within a decentralized primary healthcare network lacking a dedicated ERP system. The data used involved procurement and inventory records extracted from the supplier’s ERP system, covering the period from 2016 through 2024. The research involved designing and implementing an integrated data warehouse and analytics solution using procurement data from an external supplier ERP system. In this step, supplier’s ERP data is extracted and integrated in PHCC’s EDW, using which a dimensional star schema model was implemented to deliver decision-support through a KPI-driven analytics dashboard. For demonstration, the artifact was deployed across 32 pharmacies and evaluated through real-world operational use, focusing on its ability to enhance procurement transparency, inventory monitoring, and data-driven decision-making. Evaluation step involved iterative refinement through agile sprints and stakeholder feedbacks. The overall implementation spanned multiple stages over approximately 14 months, reflecting the complexity of enterprise-level data integration in a public healthcare setting. The following subsections detail the study context, artifact design and implementation, and evaluation approach.

### Multidisciplinary Team Formation and Collaborative Approach

The first step in our methodology was the formation of a multidisciplinary team that brought together expertise from both our healthcare organization and the supplier organization. This cross-functional team included pharmacy supply chain representatives, a pharmacy leader with deep understanding of medication management workflows, pharmaceutical supply chain, and system requirements, who had extensive experience across tertiary, secondary, and primary care levels in Qatar.

It also comprised business intelligence specialists, data analysts, and visualization experts from our organization who could translate clinical and operational needs into actionable analytics solutions. The EDW team contributed database architects and ETL specialists responsible for designing and implementing the data storage infrastructure. Additionally, Clinical Information Systems (CIS) staff brought technical expertise related to our existing EHR system, including its capabilities and limitations. Finally, the supplier organization’s business intelligence team provided specialists with in-depth knowledge of their ERP system and data structures, ensuring smooth data integration and alignment with operational goals. The team established a regular meeting cadence, with weekly status meetings to track progress and address emerging challenges. Additionally, we conducted monthly stakeholder review sessions with pharmacy leadership from across the network to ensure the solution would meet frontline operational needs.

This collaborative approach was essential for several reasons. First, it ensured that both technical and clinical perspectives informed the solution design, resulting in a more comprehensive and functional system. Second, it facilitated access to detailed knowledge about the supplier’s ERP system structure, which was critical for effective data integration. Third, it fostered shared ownership of the project outcomes across organizational boundaries, promoting alignment and accountability. Finally, it enabled rapid problem-solving when integration challenges arose. To support this collaboration, the team developed a project charter that clearly defined roles, responsibilities, timelines, and expected outcomes. This charter served as a guiding document throughout the implementation process and helped maintain focus on the core objectives of enhancing medication management capabilities and enabling centralized governance.

### System Architecture and Implementation

The primary objectives of this project were to extract comprehensive procurement data from the supplier’s ERP system, develop a robust EDW to consolidate and structure the data, implement a Tableau dashboard with actionable KPIs, and ultimately enable data-driven decision making for procurement optimization.

The system architecture supporting these objectives consisted of three main components. First, the data extraction layer utilizes custom ETL (Extract, Transform, Load) processes to securely access and extract data from the supplier’s ERP system. Second, the EDW served as a centralized data repository, designed with a star schema optimized for procurement analytics. Finally, the analytics layer included Tableau dashboards that provided visual representations of KPIs along with interactive data exploration capabilities, enabling users to gain insights and make informed decisions efficiently (Figure 3).

## Implementation Methodology

### Agile Development and Feedback Integration

**Figure 4:**
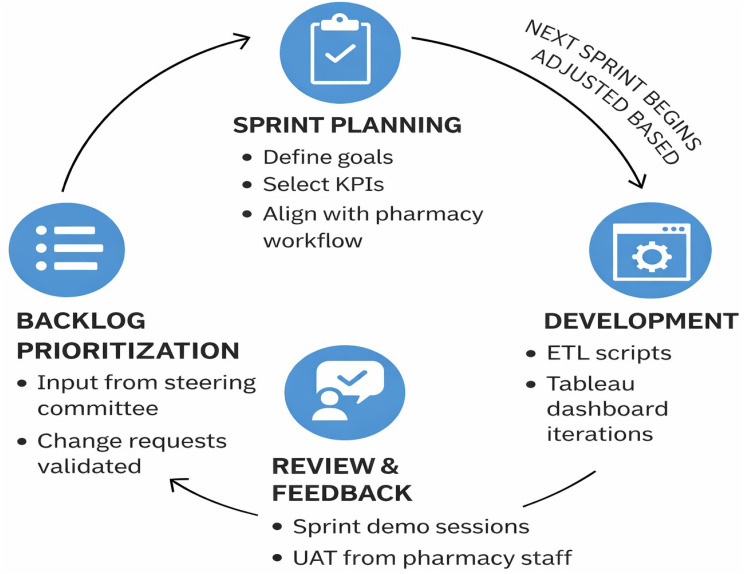
Agile Sprint Cycle and Stakeholder Feedback Integration.

To ensure a responsive and user-centered implementation, the project adopted an Agile development methodology with iterative sprint cycles (Figure 4). This approach enabled progressive enhancements to the dashboard and continuous stakeholder engagement, aligning with best practices for implementing business intelligence in healthcare environments.^25^

Each sprint lasted two to three weeks and began with goal-setting meetings that included representatives from EDW, BI, pharmacy supply chain, pharmacy operations, and the supplier’s BI and EDW teams. Cross-functional collaboration allowed the team to synchronize technical implementation with pharmaceuticals supply workflows, echoing ERP integration strategies.^26^

At the end of each sprint, live review sessions were held with stakeholders from four PHCC pharmacies. Feedback was collected using structured forms and open discussions, then integrated into the development backlog. For instance, user input led to the separation of KPIs for cancelled versus back-ordered items and the addition of interactive drill-downs to transaction-level data.

A project steering committee met regularly and communicate frequently via e-mails to validate changes, define KPIs, and oversee data quality assurance processes. This governance structure was crucial for maintaining alignment with operational needs and strategic objectives, consistent with the success factors.^19^

User acceptance testing (UAT) was integrated into each sprint cycle, ensuring that dashboard terminology, logic, and workflows were aligned with user expectations before production release. This iterative validation process helped foster user trust and adoption, addressing critical ERP post-implementation challenges.^14^

The project’s implementation was organized into four structured phases, each building on the outcomes of the previous one:

#### Phase 1: Data Assessment and Extraction Design

The initial phase focused on understanding the structure and availability of data within the supplier’s ERP system. This phase spanned approximately five months. The project team worked collaboratively to:

- Identify critical data elements essential for pharmaceutical procurement analysis, including item codes, transaction dates, lot numbers, expiration dates, order quantities, and delivery statuses.
- Define historical and real-time data ranges, with the aim of extracting procurement records dating back to 2016 and enabling near real-time updates for ongoing monitoring.
- Develop secure API connections to interface with the supplier’s ERP, ensuring data access complied with cybersecurity and governance protocols.
- Design and schedule data extraction procedures, including incremental refreshes and batch processing logic to avoid system overload and maintain data integrity.

#### Phase 2: Enterprise Data Warehouse (EDW) Development

With the extracted data available, the second phase involved building a centralized data repository optimized for analytics, a process that spanned approximately eight months:

- A dimensional data model was designed using a star schema structure to facilitate efficient querying and visualization, tailored specifically to the needs of pharmaceutical procurement.
- Data validation and quality assurance routines were implemented to handle inconsistencies in product naming, units of measure, and transaction timestamps.
- ETL (Extract, Transform, Load) processes were developed and automated to ensure reliable transformation and loading of data into the EDW, maintaining consistency between the source ERP system and the analytical environment.

This centralized EDW provided a robust and scalable infrastructure for supporting performance dashboards and enterprise reporting.

#### Phase 3: Dashboard Development and KPI Design

The project team translated the data into actionable insights through the development of interactive dashboards, which was completed over approximately two months:

- Key Performance Indicators (KPIs) were defined collaboratively with procurement stakeholders to ensure alignment with operational and strategic goals. Metrics such as perfect order rate, back-order quantity, shelf life, and return rates were prioritized.
- Interactive dashboards were built in Tableau, incorporating dynamic filters, region-level segmentation, and time-based comparisons.
- Drill-down functionality and custom visualizations were implemented to allow users to move from summary metrics to detailed transaction records, enhancing decision-making at both tactical and executive levels.

The dashboards served as the primary interface for end-users to access and explore procurement insights across all 32 pharmacies.

#### Phase 4: Validation, Optimization, and Deployment

The final phase ensured the solution was functional, efficient, and ready for organizational rollout, which was completed over approximately one month:

- User Acceptance Testing (UAT) was conducted with pharmacy supply chain coordinators and operational leads to verify dashboard logic, terminology, and workflow alignment.
- Performance optimization techniques including data aggregation, query tuning, and caching were applied to ensure fast and stable dashboard responsiveness.
- Upon completion, the system was deployed to production, and targeted training sessions were delivered to pharmacy users to promote adoption and long-term sustainability.

## Key Performance Indicators

The dashboard implementation focused on several critical KPIs for pharmaceutical procurement, providing comprehensive visibility across the supply chain:

1. Ordered Items: Total number of unique medication items ordered across all pharmacies
2. Ordered Quantity: Aggregate volume of medications ordered in standard units
3. Shipped Quantity: Actual volume of medications delivered to pharmacies
4. Returned Quantity: Volume of medications returned to the supplier due to quality issues, incorrect orders, or other reasons
5. Back Order Quantity: Volume of medications ordered but not fulfilled due to supply constraints
6. Perfect Orders: Orders delivered complete, on time, and with correct documentation
7. Perfect Order Rate: Percentage of orders that meet all perfect order criteria
8. On-time Orders: Orders delivered within agreed timeframes
9. On-time Delivery Rate: Percentage of orders that arrived on schedule
10. Return Rate: Percentage of medications delivered and returned to the supplier
11. Shipped Amount (QR): Financial value of shipped medications
12. Back Ordered Amount (QR): Financial value of medications on back order
13. Average Delivery (days): Mean time between order placement and receipt
14. Average Shelf Life (days): Mean remaining shelf life of received medications
15. Cancelled Orders: Number of orders cancelled after initial placement
16. Cancelled Items: Number of unique medication items affected by cancellations

**Figure 5:**
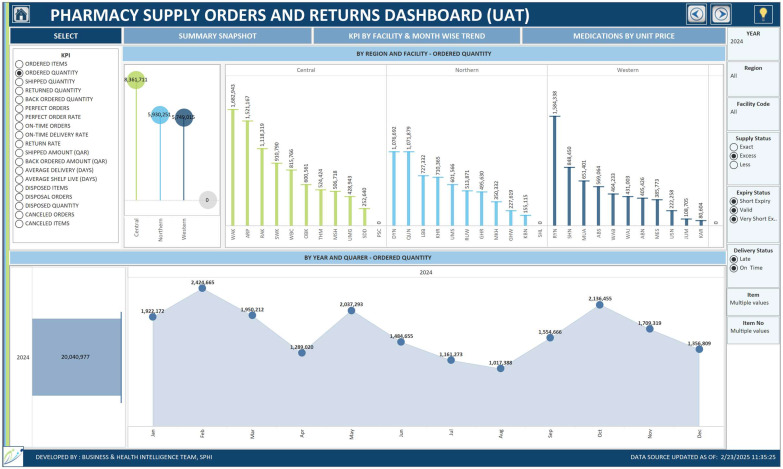
The Dashboard KPIs Page.

These KPIs were visually represented through interactive charts, allowing users to drill down from high-level metrics to detailed transaction data across the 32 pharmacy locations. The dashboard enabled filtering by time period, pharmacy location, medication category, and supplier, providing customized views for different stakeholder needs. (Figure 5)

### Challenges and Solutions

Key significant challenges were encountered during implementation presented in (Table 1).

## Results

**Figure 6:**
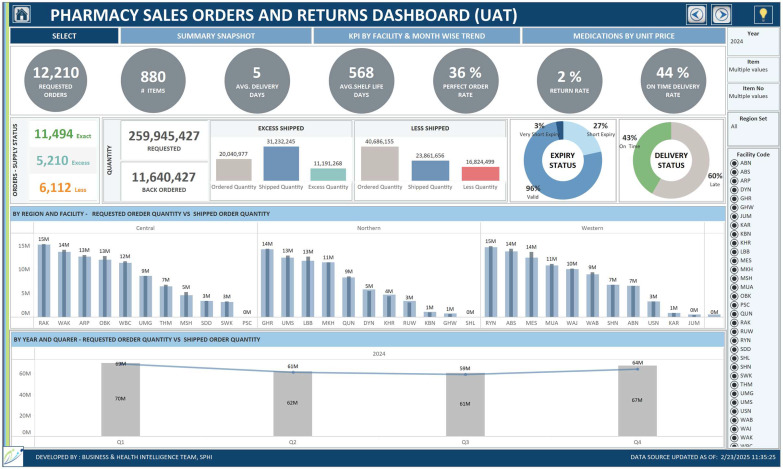
The Dashboard Summary Page for the Data from 2016 to 2024.

**Figure 7:**
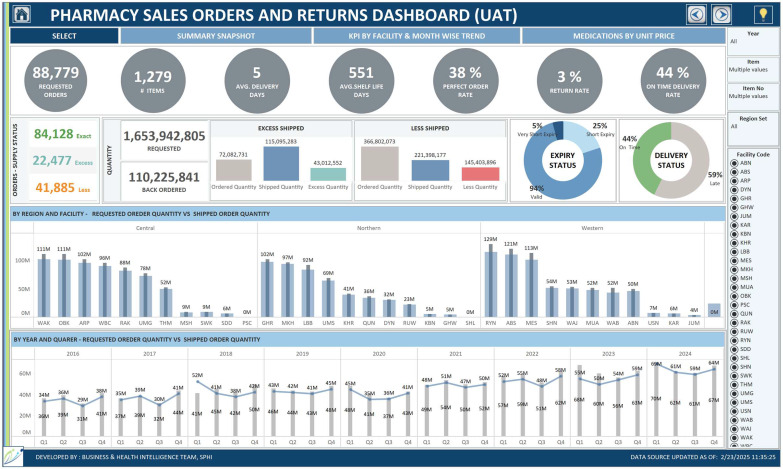
The Dashboard Summary Page for the Data of 2024.

The implementation of the integrated data warehouse and analytics dashboard yielded substantial improvements in procurement visibility and operational efficiency across our network of 32 pharmacies. The dashboard summary page (Figures 6 and 7) provides a comprehensive overview of key performance metrics and trends.

### Key Performance Metrics

Analysis of the dashboard data revealed the following performance metrics:

- Order Volume: The system processed 88,779 requested orders historically across 1,279 unique medication items, with recent 2024 data showing 12,210 requested orders across 880 unique items. This indicates a more focused procurement approach with fewer unique items being managed.
- Delivery Performance: The average delivery time has remained consistent at 5 days, with the on-time delivery rate maintaining at 44% in both historical and current data.
- Inventory Management: The average shelf life of medications improved from 551 days historically to 568 days in 2024, representing a 3% increase. The inventory expiry profile also improved significantly, with current data showing 96% valid inventory, 27% with short expiry, and only 3% with very short expiry, compared to historical figures of 25% short expiry and 5% very short expiry.
- Order Fulfilment: The perfect order rate decreased slightly from 38% historically to 36% in 2024. Current data shows 11,494 exact-match orders, with 5,210 excess-quantity orders and 6,112 under-shipped orders, providing detailed insights for order accuracy improvement initiatives.
- Financial Impact: The dashboard tracked financial dimensions across time periods, with historical data showing 1,653,942,805 requested units and 110,225,841 back-ordered units (6.7% back-order rate). The 2024 data shows improvement with 259,945,427 requested units and 11,640,427 back-ordered units, representing a reduced back-order rate of approximately 4.5%.
- Returns Management: The return rate improved from 3% historically to 2% in 2024, indicating enhanced supplier quality and order accuracy.

### Regional Analysis

The dashboard revealed significant variations in procurement patterns across different regions and facilities. In the Central Region, ordering patterns were generally balanced, with some facilities such as WAK, OBK, and ARP consistently processing larger order volumes, historically reaching approximately 100–111 million units. In 2024, however, RAK emerged as the leading facility with 15 million units, followed by WAK with 14 million and ARP with 12 million, indicating shifts in internal ordering dynamics. The Northern Region displayed moderately high procurement volumes, with GHR historically managing the largest share at approximately 102 million units. The 2024 data confirmed GHR’s continued prominence with 14 million units, followed closely by UMS and LBB, each with 13 million units. The Western Region historically exhibited the highest overall procurement volumes, with MES processing around 129 million units. However, the 2024 data showed a more balanced distribution, as RYN facility surpassed MES with 15 million units compared to MES’s 14 million, suggesting evolving utilization patterns. Overall, the 2024 regional data reflected a more balanced procurement distribution across all three regions, indicating progress toward standardizing procurement practices across the healthcare network.

### Temporal Trends

Year-over-year analysis revealed consistent growth in procurement volumes from 2016 through 2024, accompanied by notable seasonal patterns. Historically, regular quarterly fluctuations were observed, with typically higher volumes in the first and third quarters. A substantial increase in procurement volume became evident starting in 2021, marking a shift in overall demand levels. The 2024 data, in particular, demonstrated more stable quarterly procurement volumes ranging from 69 to 70 million units in Q1, 61 to 62 million in Q2, 59 to 61 million in Q3, and 64 to 67 million in Q4 indicating the emergence of more predictable ordering patterns compared to previous years. The highest volume quarters in 2024 were Q1, with 70 million units, and Q4, with 67 million units, which align with historical seasonal trends but show less pronounced fluctuations, reflecting improved forecasting and procurement stability.

### Operational Benefits

The integration of the data in PHCC Enterprise Data Warehouse (EDW) filled the gaps between the partner ERP system and the Cerner EHR system. For example, this allowed us to know Cost of the medication, Expiry Date, Lot Number (Batch Number) etc. This integration helped achieve a single pane of data.

Decision-making was also enhanced, with data-driven inventory optimization leading to more effective management of products nearing expiration. This was reflected in an increase in average shelf life to 568 days and 96% valid inventory in 2024. Operational efficiency improved through comparative regional analysis, which identified facilities with excess ordering or supply issues and resulted in a more balanced distribution across regions in 2024. Financial control was strengthened by better tracking of requested versus shipped quantities, contributing to a reduction in the back-order rate from 6.7% historically to 4.5% in 2024. Finally, the system enabled ongoing quality improvement by monitoring return rates and perfect order rates, which established baseline metrics for continuous improvement initiatives and led to a reduction in the return rate from 3% to 2%.

## Discussion

This project demonstrates the significant value that can be derived from integrating external supplier data into a healthcare organization’s analytics ecosystem. The successful implementation required a combination of technical expertise, domain knowledge, and careful stakeholder management across our network of 32 pharmacies. The findings align with prior studies emphasizing user involvement, governance alignment, and dashboard-driven performance monitoring as critical to healthcare supply chain success.^12^,^18^,^19^ As endorsed by recent empirical studies on the benefits of healthcare digitalization, our findings reinforce the positive impact of digitally-enabled supply chain systems.^12^,^19^,^27^

## Impact on Pharmaceutical Supply Chain Management

Our implementation, while not yielding measurable gains in operational KPIs, closely mirrors call for integrated inventory solutions.^7^ By linking supplier ERP data to our enterprise warehouse and layering on a Tableau dashboard, we delivered a unified, visually intuitive platform that makes tracking across our 32 pharmacies comprehensible and fully transparent, enabling stakeholders at all levels to monitor procurement status, lot batch details, and expiration data in real time.

Our approach to inventory management, particularly regarding expiry date tracking, supports the inventory optimization strategies outlined by Ahmadi et al^10^ for healthcare supplies. The significant improvement in valid inventory (96% in 2024) and reduction in very short expiry products (from 5% to 3%) reflects successful implementation of these principles. The results suggest implementing integrated EDW architectures with stakeholder-driven KPI design, phased rollout, continuous dashboard refinement, and balanced scorecard-aligned supply chain strategies can enhance transparency, governance, and inventory performance across healthcare systems.

## Business Intelligence Implementation of the Dashboard

Our findings align with research by Foshay et al,^25^ who proposed an implementation framework for business intelligence in healthcare that emphasizes the importance of organizational and cultural factors The multidisciplinary team approach we employed was instrumental in achieving adoption across diverse stakeholder groups and addressing the unique challenges of healthcare data integration.

The implementation of interactive dashboards with drill-down capabilities exemplifies the principles described by for hospital quality dashboards.^18–20^ Their research highlighted the importance of visual presentation, user-centered design, and organizational embedding, all factors that contributed to the successful adoption of our system across 32 pharmacy locations.

## Technical Approach and System Architecture

The technical approach we followed is consistent with ERP assimilation models proposed by Shao et al^28^ that highlight the importance of leadership styles and organizational learning. As noted by Nwankpa et al^29^ successful ERP implementation requires careful consideration of system usage factors, which was a central focus of our project. Our ability to process and analyze large procurement datasets (over 88,000 orders with 1,279 unique items historically, and 12,210 orders with 880 items in 2024) demonstrates effective integration of external ERP data.

The data warehouse design implemented in our project follows the principles outlined by Ferranti et al^30^ for leveraging business intelligence tools in healthcare settings. Their emphasis on integrating financial and clinical data for improved decision-making is reflected in our dashboard’s ability to connect order fulfillment metrics (perfect order rate, on-time delivery rate) with inventory management outcomes (shelf life, expiry profiles).

## Performance Measurement and KPI Selection

Our dashboard development strategy incorporated balanced scorecard principles, while also leveraging information-sharing approaches described by for business intelligence systems. The selection of 19 distinct KPIs focused on order processing, fulfillment accuracy, delivery performance, and inventory quality aligns with best practices for healthcare supply chain measurement.^31^

The regional analysis capabilities we implemented, which revealed significant variations in procurement patterns across our three regions, support the findings of Stefanou et al^15^ and Bialas et al^16^ regarding the importance of contextual analysis in healthcare ERP integration. Our ability to identify facilities with the highest procurement volumes (RAK, RYN, and GHR in 2024) enabled targeted process improvement initiatives.

### Agile Development Approach

The adoption of an Agile development approach was instrumental in ensuring the success and scalability of the dashboard across PHCC’s network. Unlike traditional project management methods, the Agile model allowed for continuous refinement based on frontline feedback, reducing resistance to adoption and enabling a tailored solution that met real-world clinical and procurement needs.

The effectiveness of this methodology is evident in several outcomes. First, the phased and interactive development process fostered a sense of ownership among end users, enhancing adoption rates across decentralized pharmacy sites. This supports the findings of Foshay et al,^25^ who emphasized the value of cultural alignment and iterative delivery in business intelligence initiatives.

Second, the project’s governance structure anchored in the project steering committee proved vital in sustaining data quality, resolving KPI disputes, and prioritizing dashboard enhancements. As noted by Helminski et al^19^ and Coiera et al^18^ dashboards achieve maximum value when supported by both operational relevance and executive oversight.

Third, the consistent feedback loops between users and developers directly influenced performance optimization. Early feedback on slow rendering times led to implementation of materialized views and query pre-aggregation, improving dashboard responsiveness. These refinements reflect the principles of healthcare dashboard performance.^27^

Overall, the use of Agile methodology not only shaped the technical development of the system but also enhanced organizational learning, data governance, and stakeholder alignment key pillars for successful digital transformation in healthcare, as outlined in ERP and BI literature.^28^

### Success Factors and Lessons Learned

Several key success factors contributed to the effectiveness of our implementation. First, cross-organizational collaboration with the supplier organization played a vital role by enabling secure data access and seamless system integration. This partnership was crucial in overcoming the technical complexities associated with external data extraction. Second, a user-centered design approach through the active involvement of procurement specialists in defining KPIs and shaping the dashboard ensured that the final product was both relevant and widely adopted. Third, a phased, incremental implementation allowed for continuous feedback and refinement, resulting in progressively improved KPIs, as reflected in the enhanced performance metrics observed in 2024. Fourth, a strong focus on system performance supported user adoption, aligning with best practices recommended by Kandy et al^27^ for healthcare dashboard implementation. Fifth, robust data quality governance, including clear validation and assurance processes, was essential in maintaining user trust, particularly for critical metrics such as expiration dates and perfect order rates.

The implementation of the dashboard successfully transformed previously fragmented pharmacy operations into a unified, data-driven network with standardized performance measurement and enhanced corporate oversight. By offering both high-level summary metrics and detailed drill-down views by region, facility, and time period, the system supported strategic planning and enabled targeted, tactical interventions to optimize the pharmaceutical supply chain. The evolution of results from initial implementation to current performance in 2024 demonstrates the value of continuous improvement in healthcare analytics. The observed improvements in regional procurement distribution, reduced return rates, and increased inventory validity underscore both the maturity of the system and the growth in organizational capability.

## Future Work

Building on the success of this implementation, several enhancements are planned to further strengthen the system’s capabilities and support more advanced decision-making. First, the introduction of predictive analytics through machine learning models will enhance demand forecasting, leveraging the potential of big data analytics. Second, expanded integration efforts will include additional supplier systems and internal clinical data, creating a more comprehensive and interconnected data ecosystem. Third, the development of mobile interfaces will provide on-the-go access to decision support tools, improving responsiveness and accessibility for stakeholders. Fourth, an automated alert system based on threshold-triggered notifications will be implemented to flag procurement exceptions, drawing inspiration from cybersecurity monitoring strategies.^32^ Lastly, advanced dashboard enhancements will incorporate sophisticated data visualization techniques, in line with the methods outlined by, Helminski et al^19^ and Coiera et al^18^ to further improve user engagement and interpretability of complex data trends.

Key limitations of this study are the absence of a statistical or inferential evaluation and reliance on a single-case, observational assessment of improvements. Future work should apply rigorous quantitative impact analysis with properly designed before–and–after statistical testing across multiple healthcare networks to strengthen generalizability and causal evidence. Additionally, incorporating a control or comparison case in the network would help isolate the true impact of the implemented changes and improve causal attribution.

## Conclusion

This case study has demonstrated the critical value of integrating pharmaceutical procurement data from an external supplier’s ERP system into PHCC enterprise-wide data architecture, supported by a dynamic Tableau-based analytics platform. Through a structured and collaborative implementation process, we achieved a centralized system that significantly enhanced transparency, traceability, and governance across Qatar’s primary healthcare pharmaceutical supply chain. The transformation from a fragmented, manually monitored procurement process into a unified, data-driven environment represents a major advancement in supply chain maturity within the public healthcare sector. This study further contributes to the literature by demonstrating how integrated data and dimensional modelling and KPI-driven dashboards can directly enhance inventory expiry control, reduce back-order rates, and strengthen financial oversight within a multi-pharmacy network.

The project yielded measurable benefits across several operational dimensions. Beyond the technical achievements, the success of this initiative hinged on key organizational enablers: strong cross-functional collaboration, user-centered design, and iterative deployment cycles. These factors, supported by a clear governance structure and continuous stakeholder engagement, fostered trust in the system and widespread adoption across 32 decentralized pharmacy sites. Importantly, the project serves as a replicable model for healthcare organizations operating without a centralized ERP system.

Looking forward, the roadmap for future enhancements, including predictive analytics, mobile access, integration with clinical data, and intelligent alert systems positions the platform to evolve into a comprehensive digital ecosystem. These capabilities will not only support advanced procurement forecasting and exception management but also contribute to broader health system goals such as cost containment, supply chain resilience, and patient safety.

In conclusion, this initiative has not only bridged the gap between clinical service delivery and backend logistics but has also laid the groundwork for a data-intelligent healthcare supply chain in Qatar. It underscores the potential of targeted digital transformation to elevate operational performance and governance in complex, resource-sensitive environments. Other healthcare institutions can draw valuable insights from this model to pursue similar gains in efficiency, quality, and strategic oversight in their supply chain operations.

## Generative Artificial Intelligence

Generative Artificial Intelligence tools (ChatGPT, GPT-5, OpenAI, 2025 version) were used to assist in language refinement and structural editing of this manuscript. The author reviewed, verified, and is fully responsible for all content and interpretations presented.

## References

[1] Adhikari B, Ranabhat K, Khanal P, et al. Procurement process and shortages of essential medicines in public health facilities: a qualitative study from Nepal. *PLOS Global Public Health*. 2024;4(5):e0003128. doi:10.1371/journal.pgph.0003128 38696399 PMC11065305

[2] Abdel Rida N, Izham M, Zaidan M. An exploratory insight on pharmaceutical sector and pricing policies in Qatar. *Global J Pharma Pharmaceu Sci*. 2017;1(4). doi:10.19080/GJPPS.2017.01.555568

[3] Oxford Business Group. Qatar health care industry introduces policy updates, seeks FDI - Qatar 2024 - oxford business group. 2024. Available from: https://oxfordbusinessgroup.com/reports/qatar/2024-report/health/shifting-focus-policy-updates-aim-to-galvanise-the-domestic-health-care-industry-and-attract-foreign-investment-overview/. Accessed May 17, 2025.

[4] AlZaidan M, Hadid M, Padmanabhan R, Kerbache L. Optimizing pharmaceutical supply chain configuration in primary healthcare: a mathematical modeling and decision support approach. In: Proceedings of the International Workshop on Innovative Simulation for Health Care, IWISH, Vols 2024-September. Cal-Tek srl; 2024. doi:10.46354/i3m.2024.iwish.008.

[5] MOPH. National Health Strategy. 2024-2030. Health for All. 2024. Available from: https://www.moph.gov.qa/english/NHS/Pages/default.aspx. Accessed May 3, 2025.

[6] Settanni E, Harrington TS, Srai JS. Pharmaceutical supply chain models: a synthesis from a systems view of operations research. *Operations Res Perspect*. 2017;4:74–20. doi:10.1016/j.orp.2017.05.002

[7] Rajabi R, Shadkam E, Khalili SM. Design and optimization of a pharmaceutical supply chain network under COVID-19 pandemic disruption. *Sustainable Operations Computers*. 2024;5:102–111. doi:10.1016/j.susoc.2024.04.002

[8] Kelle P, Woosley J, Schneider H. Pharmaceutical supply chain specifics and inventory solutions for a hospital case. *Oper Res Health Care*. 2012;1(2–3):54–63. doi:10.1016/j.orhc.2012.07.001

[9] Fernandez MI, Chanfreut P, Jurado I, Maestre JM. A data-based model predictive decision support system for inventory management in hospitals. *IEEE J Biomed Health Inform*. 2021;25(6):2227–2236. doi:10.1109/JBHI.2020.3039692 33216723

[10] Ahmadi E, Masel DT, Metcalf AY, Schuller K. Inventory management of surgical supplies and sterile instruments in hospitals: a literature review. *Health Systems*. 2019;8(2):134–151. doi:10.1080/20476965.2018.1496875 31275574 PMC6598505

[11] Batz A, D’Croz-Barón DF, Vega Pérez CJ, Ojeda-Sanchez CA. Integrating machine learning into business and management in the age of artificial intelligence. *Humanit Soc Sci Commun*. 2025;12(1):352. doi:10.1057/s41599-025-04361-6

[12] Shahzad K, Khan SA, Latif M, Javeed AMD, Iqbal A. Big data analytics in healthcare: current practices, innovations, and future prospects. *J Big Data*. 2025;12(1):242. doi:10.1186/s40537-025-01288-2

[13] Al-Assaf K, Alzahmi W, Alshaikh R, Bahroun Z, Ahmed V. The relative importance of key factors for integrating enterprise Resource Planning (ERP) systems and performance management practices in the UAE healthcare sector. *Big Data Cognitive Computing*. 2024;8(9):122. doi:10.3390/bdcc8090122

[14] Butarbutar ZT, Handayani PW, Suryono RR, Wibowo WS. Systematic literature review of Critical success factors on enterprise resource planning post implementation. *Cogent Business Manage*. 2023;10(3). doi:10.1080/23311975.2023.2264001

[15] Stefanou CJ, Revanoglou A. ERP integration in a healthcare environment: a case study. *J Enterprise Informat Manage*. 2006;19(1):115–130. doi:10.1108/17410390610636913

[16] Bialas C, Bechtsis D, Aivazidou E, Achillas C, Aidonis D. Digitalization of the healthcare supply chain through the adoption of Enterprise Resource Planning (ERP) systems in hospitals: an empirical study on influencing factors and cost performance. *Sustainability*. 2023;15(4):3163. doi:10.3390/su15043163

[17] Ghazisaeidi M, Safdari R, Torabi M, Mirzaee M, Farzi J, Goodini A. Development of performance dashboards in healthcare sector: key practical issues. *Acta Informatica Medica*. 2015;23(5):317. doi:10.5455/aim.2015.23.317-321 26635442 PMC4639357

[18] Coiera E, Chan A, Brooke-Cowden K, Rahimi-Ardabili H, Halim N, Tufanaru C. Clinical and economic impact of digital dashboards on hospital inpatient care: a systematic review. *JAMIA Open*. 2025;8(4). doi:10.1093/jamiaopen/ooaf078 PMC12296400 40718761

[19] Helminski D, Sussman JB, Pfeiffer PN, et al. Development, implementation, and evaluation methods for dashboards in health care: scoping review. *JMIR Med Inform*. 2024;12:e59828. doi:10.2196/59828 39656991 PMC11651422

[20] Lemak CH, Pena D, Jones DA, Kim DH, Guptill J. Leadership to accelerate healthcare’s digital transformation: evidence from 33 health systems. *J Healthcare Manage*. 2024;69(4):267–279. doi:10.1097/JHM-D-23-00210 38976787

[21] Polimeno A, Braghin C, Anisetti M, Ardagna CA. Maximizing data quality while ensuring data protection in service-based data pipelines. *J Big Data*. 2025;12(1):62. doi:10.1186/s40537-025-01118-5 38603580

[22] Gobachew AM, Haasis H-D, Berhan E. Case assessment and identification of pharmaceutical supply chain performance measures and metrics. *African J Sci Technol Innovat Develop*. 2023;15(4):524–531. doi:10.1080/20421338.2022.2153981

[23] Pejić Bach M, Klinčar A, Aleksić A, Rašić Jelavić S, Zeqiri J. Supply chain management maturity and business performance: the balanced scorecard perspective. *Appl Sci*. 2023;13(4):2065. doi:10.3390/app13042065

[24] Hjelle S, Mikalef P, Altwaijry N, Parida V. Organizational decision making and analytics: an experimental study on dashboard visualizations. *Informat Manage*. 2024;61(6):104011. doi:10.1016/j.im.2024.104011

[25] Foshay N, Kuziemsky C. Towards an implementation framework for business intelligence in healthcare. *Int J Inf Manage*. 2014;34(1):20–27. doi:10.1016/j.ijinfomgt.2013.09.003

[26] Farzandipur M, Jeddi F, Azimi E. Factors affecting successful implementation of hospital information systems. *Acta Informatica Medica*. 2016;24(1):51. doi:10.5455/aim.2016.24.51-55 27041811 PMC4789654

[27] Kandy MC, Abdulmajeed J, Gohel CN, Gibb JM, Al-Kuwari MG. Transforming primary healthcare services with centralized health intelligence: a case study from Qatar. *Qatar J Public Health*. 2024;2023(2). doi:10.5339/qjph.2023.8

[28] Shao Z, Feng Y, Hu Q. Impact of top management leadership styles on ERP assimilation and the role of organizational learning. *Informat Manage*. 2017;54(7):902–919. doi:10.1016/j.im.2017.01.005

[29] Nwankpa JK. ERP system usage and benefit: a model of antecedents and outcomes. *Comput Human Behav*. 2015;45:335–344. doi:10.1016/j.chb.2014.12.019

[30] Ferranti JM, Langman MK, Tanaka D, McCall J, Ahmad A. Bridging the gap: leveraging business intelligence tools in support of patient safety and financial effectiveness. *J Ame Med Informat Assoc*. 2010;17(2):136–143. doi:10.1136/jamia.2009.002220 PMC3000785 20190055

[31] Popovič A, Hackney R, Coelho PS, Jaklič J. How information-sharing values influence the use of information systems: an investigation in the business intelligence systems context. *J Strategic Informat Syst*. 2014;23(4):270–283. doi:10.1016/j.jsis.2014.08.003

[32] Radanliev P, De Roure D, Page K, et al. Cyber risk at the edge: current and future trends on cyber risk analytics and artificial intelligence in the industrial internet of things and industry 4.0 supply chains. *Cybersecurity*. 2020;3(1):13. doi:10.1186/s42400-020-00052-8

